# Rapid Discrimination of *Citrus reticulata* ‘Chachi’ by Electrospray Ionization–Ion Mobility–High-Resolution Mass Spectrometry

**DOI:** 10.3390/molecules26227015

**Published:** 2021-11-20

**Authors:** Juan Liu, Keke Wang, Yuling Li, Bowen Zhou, Kuofeng Tseng, Xiaoqiang Zhang, Yue Su, Wenjian Sun, Yinlong Guo

**Affiliations:** 1 Center for Chinese Medicine Therapy and Systems Biology, Institute for Interdisciplinary Medicine Sciences, Shanghai University of Traditional Chinese Medicine, 1200 Cailun Road, Shanghai 201203, China; liujuan@sioc.ac.cn; 2 National Center for Organic Mass Spectrometry in Shanghai, Shanghai Institute of Organic Chemistry, Chinese Academy of Sciences, 345 Lingling Road, Shanghai 200032, China; liyl@sioc.ac.cn (Y.L.); zhoubowen@sioc.ac.cn (B.Z.); 3 Shimadzu Research Laboratory (Shanghai) Co., Ltd., Shanghai 201206, China; wangkeke@srlab.com.cn (K.W.); tsengkuofeng@srlab.com.cn (K.T.); zhangxiaoqiang@srlab.com.cn (X.Z.)

**Keywords:** ion mobility-quadrupole time of flight mass spectrometry, *Citri reticulatae* pericarpium, *Citrus reticulata* ‘Chachi’, polymethoxylated flavones, isomers

## Abstract

A common idea is that some dishonest businessmen often disguise *Citrus reticulata* Blanco varieties as *Citrus reticulata* ‘Chachi’, which places consumers at risk of economic losses. In this work, we combined high-resolution ion mobility (U-shaped mobility analyzer) with high-resolution mass spectrometry to rapidly distinguish *Citrus reticulata* ‘Chachi’ from other *Citrus* species. The samples were analyzed directly through simple extraction and the analytes were separated in one second. It only took about 1 min to perform a cycle of sample analysis and data acquisition. The results showed that polymethoxylated flavones and their isomers were separated easily by the ion mobility analyzer and preliminarily identified according to the accurate mass. Moreover, the collision cross-section values of all analytes, which could be used as auxiliary parameters to characterize and identify the compounds in the samples, were measured. Twenty-four samples were grouped as two clusters by multivariate analysis, which meant that *Citrus reticulata* ‘Chachi’ could be effectively differentiated. It was confirmed that the developed method had the potential to rapidly separate polymethoxylated flavones and distinguish between *Citrus reticulata* ‘Chachi’ and other *Citrus reticulata* Blanco varieties.

## 1. Introduction

*Citri reticulatae* pericarpium (CRP) is traditional Chinese food medicine, which derives from the dry and ripe peel of *Citrus reticulata* Blanco or its cultivars. The original CRP plants listed in the Pharmacopoeia of the People’s Republic of China mainly include *C. reticulata* ‘Chachi’, *C. reticulata* ‘Dahongpao’, *C. reticulata* ‘Unshiu’, and *C. reticulati* ‘Tangerina’. The peel is harvested, split into three pieces, and dried in the sun [1]. *C. reticulata* ‘Chachi’ produced in Xinhui, China (called “Guangchenpi”, GCP) is considered as a pre-eminent geoherb exhibiting a superb quality and high efficacy [2]. Due to its aroma and utility, GCP is commonly used to make soups, sweetmeats, snacks, and teas, such as ‘Spicy Orange Beef’, ‘Ganpu Tea’, and ‘Tangerine Power’ [3,4]. However, CP (other varieties called “Chenpi”, CP) struggles to maintain the appealing characteristics of GCP. The commercial value of CP is far less than that of GCP. But the frequent phenomenon that CP is a fake of GCP by some greedy businessmen to gain high but illegal profit has been banned repeatedly. Thus, there is an urgency to establish a simple, efficient, and reliable method to distinguish between GCP and CP.

It is hard to distinguish between GCP and CP correctly for consumers, placing them at risk of economic losses. Macroscopical identification is a traditional method of identifying GCP that is based on materials, texture, appearance, size of section characteristics, smell, and color. During identification, the method requires the rich experience of the discriminator rather than advanced and costly instruments; this is a fast, convenient, and widely used method. Hence, macroscopical identification is very popular among people who trade constantly in the market. Nevertheless, this form of identification has several noticeable drawbacks: well-experienced specialists are necessary and the most personal judgments are extremely subjective. Therefore, a unified, clear, and quantitative standard to help average consumers distinguish between GCP and CP is necessary. Recently, it was reported that various techniques were applied to identify GCP, such as electronic nose [5], electronic tongue [6], near-infrared spectroscopy [7], DNA brocade [8], or a combination of those methods. Moreover, fingerprint methodology and the metabolomics approach were also applied to identify GCP [9,10]. Additionally, recent studies reported that the chemical components of GCP mainly included volatiles, flavonoids, alkaloids, and phenolic acids [11,12,13,14]; most studies concentrated on volatiles [15,16] and flavonoids [17,18,19,20]. Rich in volatiles, *C. reticulata* Blanco and *C. reticulata* ‘Chachi’ were analyzed by gas chromatography coupled with mass spectrometry (GC-MS) in several laboratories over the past decades [21,22]. In addition to the volatiles [23], the flavonoids in GCP and CP were also analyzed by liquid chromatography [24,25,26]. Rapid resolution liquid chromatography-electrospray ionization tandem mass spectrometry was also employed to identify a total of 41 chemical constituents in CRP [27]. Furthermore, thin-layer chromatography was adopted to identify GCP [28]. As a powerful separation technique, chromatography routinely takes dozens of minutes to complete a cycle [29]. Therefore, it is necessary to establish a rapid method to separate the compounds in GCP.

Ion mobility spectrometry (IMS) [30] is a rapid separation technique on a second timescale [31]. The mechanism involves ions driven by an electric field in a gas damping environment that have a different migration rate. The ions can be separated by their charge state, size, shape, charge position, or structural rigidity [32]. For sensitive detection, IMS is suitable for the trace detection of some volatile organic compounds, such as narcotics [33], explosives [34], chemical warfare [35], and air pollutants [36]. Since the first commercial IMS was manufactured in the 1960s, it has undergone rapid growth over the past decades and been used widely in many laboratories. Varied commercial IMS instruments were manufactured, such as the drift tube ion mobility spectrometry (DTIMS) [37], traveling wave ion mobility spectrometry (TW-IMS) [38], cyclic ion mobility spectrometry (cIMS) [39], and trapped ion mobility spectrometry (TIMS) [40]. IMS can also be used in combination with chromatography. For instance, headspace–gas chromatography–ion mobility spectrometry was performed to effectively distinguish *C. reticulata* ‘Chachi’ [41]. Even though the pre-treatment was not required, it still took more time to separate analytes by GC and IMS, respectively. Fortunately, IMS can be flexibly hyphenated with various ionization sources under atmospheric pressure. Electrospray ionization (ESI) is a soft ionization technique that has already been successfully coupled with the IM-MS instrument [42]. Moreover, IM-MS solved the problem that MS was limited for distinguishing isomeric species. The ion’s mass-to-charge ratio (*m/z*) and average collision cross-section (CCS) can be obtained, which leads to the rising popularity in many fields, including natural products [43,44], microorganisms [45], carbohydrates [46,47], lipidomics [48,49,50], proteomics [51,52], food [53], and environmental samples [54,55]. With current advances in apparatus, IMS is used as a tool in analytical and bioanalytical applications, rather than as a detector for chemical warfare agents and explosives. The recent development tendency of the ion mobility analyzer is toward a higher performance for completing the increasing measurement task complexity, especially for ultra-high resolutions (>ca. 200) [56]. The U-shaped mobility analyzer (UMA) achieved a resolution of about ca. 180 for single-charge small organic molecules, and up to ca. 370 for multiple-charge +15 myoglobin [57]. Additionally, there is an alternative strategy for identifying isomers of little difference via UMA.

In this study, the chemical composition of GCP and CP were observed, especially, isomers of polymethoxylated flavones (PMFs). Specifically, ESI transferred ions directly from the solution into the gas phase [58], and gas-phase ions were separated by IMS in one second. Then, the accurate mass could be measured simultaneously by HRMS and CCS values, which were calculated in the next step. As a result, not only was the rapid separation of ions achieved but the CCS values and accurate mass measurements were also obtained. Then, a principal component analysis (PCA) and hierarchical cluster analysis (HCA) were performed to distinguish between GCP and CP. The results of PCA showed that the samples were grouped into two sets. On the heatmap of HCA, it could be seen that GCP and CP were clustered and divided into two groups. Namely, GCP and CP could be differentiated by ESI-IM-HRMS, which could be used as an auxiliary method for the identification of medicinal materials.

## 2. Results and Discussion

### 2.1. Optimization of Sample Extraction and Instrument Settings

To raise the better extraction efficiency of PMFs, the central parameters, including extraction solvent, ultrasonic extraction time, and volume of extraction were optimized. Since PMFs contain multiple methoxy groups with low polarity and planar structure, we initially attempted to extract PMFs with low polar solvents, such as chloroform and hexane [59]. However, chloroform and hexane are not compatible with ESI so it was necessary to blow dry these substances with nitrogen and dissolve them with methanol before the analysis. Methanol was also used as extraction of PMFs in recent studies [5,18,25]. Moreover, methanol is conducive to ESI, which saved time in drying extracting solutions. In addition, 50% methanol and 70% methanol were used in other studies [27,60]. Therefore, we designed different proportions of methanol for the extraction experiments. First, HPLC-grade methanol and deionized water were mixed by methanol-water (30:70, *v/v*), methanol-water (50:50, *v/v*), methanol-water (70:30, *v/v*), and methanol. Then, ultrasonic extraction times: 30 min, 45 min, 60 min, and 90 min were used in the experiment. Amounts of 5ml, 10 mL, 20mL, and 40mL of methanol were added to the samples, respectively. Taking three strong peaks of PMFs (*m/z* 373.1271, *m/z* 403.1383, *m/z* 433.1484) as a reference, 5 mL methanol (Figure S1a,b in the Supplementary Materials) and 60 min of ultrasonic extraction time (Figure S1c in the Supplementary Materials) were preferred as the optimal extraction conditions.

In this work, the UMA was used as a newly developed analyzer with a few reported applications [61]. Among several parameters, the electric field range and the rate of counter-flow gas in the two channels had a great influence on the resolution of analytes. To evaluate the effects of the two factors, we took a pair of isomers (tangerine and sinensetin) as an example. With a fixed scan period, the experiment illustrated that the smaller the electric field range, the slower the scanning speed, and thus a higher resolution of analytes were obtained (Figure S2a in the Supplementary Material). Sharp-point spikes and an excellent separation were both observed when the electric field range of 2.0–3.0 V/mm was selected. As for the rate of the counter-flow gas, the experiment proved that the buffer gas flow rate had an impact on the resolution and intensity of analytes by changing ions’ passing percentage. A good separation of tangerine and sinensetin was achieved when the gas flow rate was set to 1 L/min (Figure S3 in the Supplementary Material). Hence, 1.0 L/min was selected as the rate of counter-flow gas flow.

After optimizing the extraction conditions and UMA parameters, we set the ESI-IM-HRMS instrument as described in Section 3.3. The feasibility of the ESI-IM-HRMS was evaluated based on the repeatability and reproducibility [41]. The repeatability was assessed according to the relative standard deviation (RSD %) of the tallest signal intensity of six replicates on the same day. The reproducibility was evaluated by the RSD of the tallest signal intensity of three replicates for 3 consecutive days. The RSDs of repeatability and reproducibility were 1.9% and 4.9%, respectively (Tables S1 and S2 in the Supplementary Materials). The test results revealed that the ESI-IM-HRMS system had a good precision.

### 2.2. Separation of Polymethoxylated Flavones and Their Isomers by ESI-IM-HRMS

**Figure 1:**
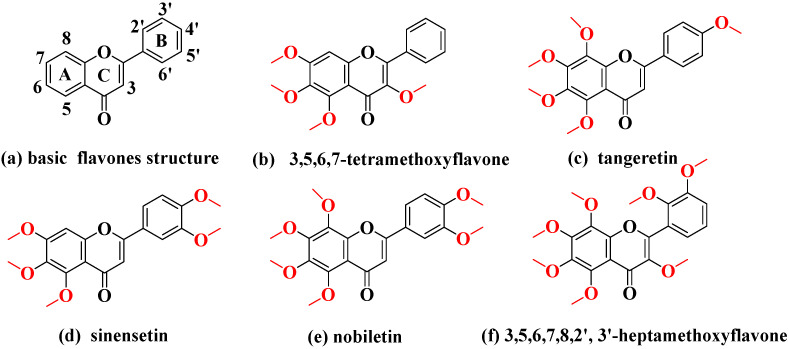
(**a**) basic structures of the flavones and chemical structures of five polymethoxylated flavones: (**b**) 3,5,6,7-tetramethoxyflavone, (**c**) tangeretin, (**d**) sinensetin, (**e**) nobiletin, (**f**) 3,5,6,7,8,2′,3′-heptamethoxyflavone.

PMFs are specific chemical components possessing antioxidant properties in the peel of *Citrus* species that have attracted researchers in recent decades [62]. There are many isomers among PMFS that are usually analyzed by chromatography. To rapidly separate PMFs in *C. reticulata* ‘Chachi’, we applied a flow-injection analysis combined with ESI-IM-HRMS. In the data processing software, there were high-resolution mass spectra and a two-dimensional (2D) heat map (Figure S4 in the Supplementary Material). The accurate mass was shown in the high-resolution mass spectra and the signal intensity of each analyte was observed by the depth of color on the heat map. Moreover, isomers could be found quickly by the spots on the 2D heat map. For instance, there were two light spots at *m/z* 373.1271 on the 2D heat map meaning that a pair of isomers were present (Figure S4a in the Supplementary Materials). After comparing the theoretically accurate mass with the measured (Table 1, relative error within 5 ppm), the compounds at *m/z* 373.1271 were identified, namely, tangeretin and sinensetin, which were positional isomers of PMFs (Figure 1c,d).

**Figure 2:**
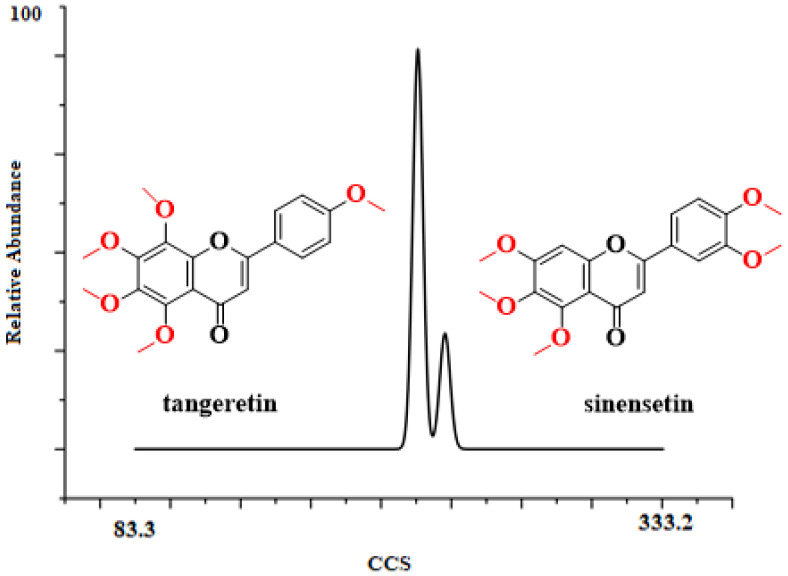
Ion mobility diagram of tangeretin and sinensetin.

It is not possible to distinguish isomers according to the mass–charge ratio. The most direct way to differentiate between them was by using the reference materials of tangeretin and sinensetin. When the UMA device was employed, it was observed that tangeretin and sinensetin could be separated very well (Figure 2). The front peak was tangeretin and the latter was sinensetin, which showed that tangeretin had a more compact structure. After software computation, the resolution of tangeretin and sinensetin were high; about 95 and 91, respectively (Figure S2a in the Supplementary Materials). Meanwhile, they only presented a tendency to be separated by DTIMS (ca. 60, Figure S5c in the Supplementary Materials). It is well-known that the resolution of IMS is associated with the ion’s effective path length. In the separation process of ions, the environment significantly affects the effective path length, electrostatic fields, electrodynamic fields, the direction of gas, and the rate of gas [31]. In DTIMS, ions pass through the drift tube where a weak uniform electric field and ambient pressure gas are present; while, in UMA, the electric field and the gas flow are in the opposite direction, which lengthens the ion’s effective path length. Compared with DTIMS, the UMA significantly upgraded the resolution of analytes. In addition, CCS values can be used to facilitate the identification of tangeretin and sinensetin, which is discussed later.

**Figure 3:**
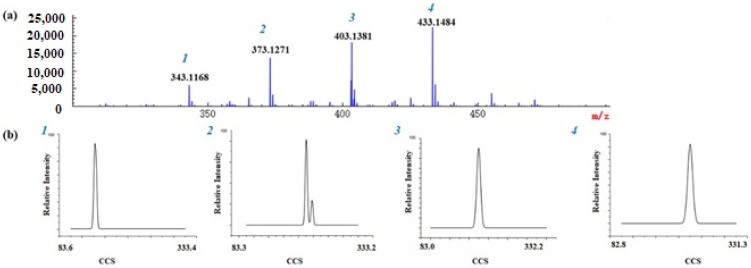
MS spectra (**a**) and UMA spectra (**b**) of five polymethoxylated flavones in Chenpi MS spectra of a, (1) 3,5,6,7-tetramethoxyflavone, (2) tangeretin and sinensetin, (3) nobiletin, (4) 3,5,6,7,8,2′,3′-heptamethoxyflavone. UMA spectra of b, (1) 3,5,6,7-tetramethoxyflavone, (2) tangeretin and sinensetin, (3) nobiletin, (4) 3,5,6,7,8,2′,3′-heptamethoxyflavone. The x-axis represents CCS values and Y-axis represents relative abundance.

MS/MS spectra are also helpful for identifying compounds based on characteristic fragment ions. PMFs tend to generate fragment ions by the loss of CH_3_^•^, H_2_O, -C=O, and so on [19]. Taking nobiletin as an example, the possible fragmentation pattern is shown in detail in Figure S6 of the Supplementary Materials. There was a protonated molecular ion at *m/z* 403.1381 [M + H]^+^ and the characteristic ions were at *m/z* 373.0913 [M + H-C_2_H_6_]^+^, 358.0677 [M + H-C_3_H_9_]^+^, 355.0805 [M + H-C_2_H_6_-H_2_O]^+^, 345.0962 [M + H-C_2_H_6_-CO]^+^, 330.0729 [M+H-C_3_H_9_-CO]^+^, 211.0230 [^1,3^A^+^-C_2_H_6_], 163.0746 [^1,3^B^+^] (Figure S7d in the Supplementary Materials). Therefore, the compound at *m/z* 403.1381 was identified as nobiletin. As above, the peaks at *m/z* 343.1168, *m/z* 403.1381 and *m/z* 433.1484 (Figure 3), were also identified as 3,5,6,7-tetramethoxyflavone, nobiletin and 3,5,6,7,8,2′,3′-heptamethoxyflavone (Figure 2b,e,f).

### 2.3. CCS Value Measurements

The main advantage of CCS values is that they enhance the specificity of the targeted screening, offer complementary orthogonal identification information, and overcome the influence of varied sample matrices, which makes these values promising in the screening and analysis of unknown compounds [31]. To obtain CCS values, the instrument conditions were set as described in Section 3.3. Unlike DTIMS, the raw data obtained by UMA-MS were built on the central electric field which could be further converted to the CCS value. The central electric fields and the accurate mass of each compound were directly measured. Then, CCS values were calculated and shown in Table 2. The CCS values of flavonoids calculated by DTIMS were widely used [63]. Comparing the CCS values of PMFs measured by UMA-HRMS with the previous studies that used DTIMS [64], it was found that the CCS values were consistently measured by the two methods (Table 3). The RSDs of CCS values were acceptable by UMA-HRMS (all less than 2%, Table S3 in the Supplementary Materials), showing that the UMA-HRMS system had a good precision for CCS value measurements.

### 2.4. ESI-IM-HRMS Analysis of GCP and CP Samples

**Figure 4:**
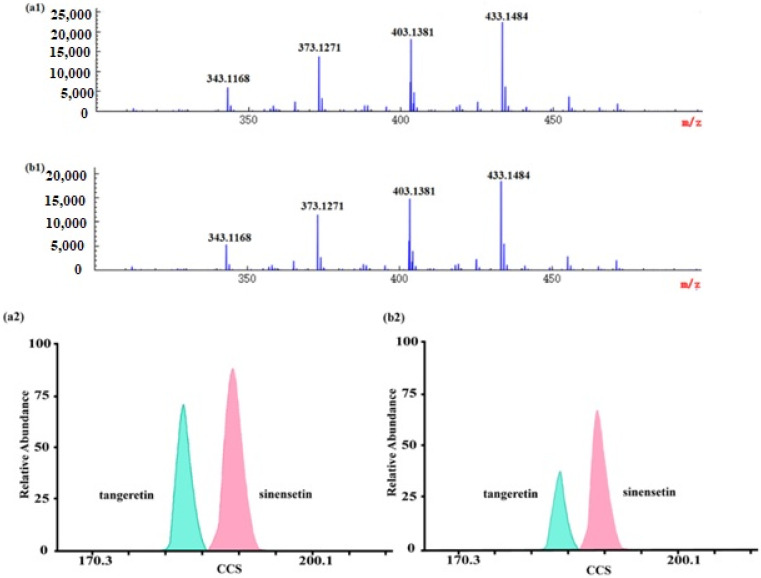
MS spectra and UMA spectra of polymethoxylated flavones in GCP and CP: (**a1**,**b1**) are MS spectra of polymethoxylated flavones in Guangchenpi and Chenpi, respectively; (**a2**,**b2**) are UMA spectra of tangeretin and sinensetin in Guangchenpi and Chenpi, respectively.

To validate the applicability of the method to the actual samples, 13 GCP and 11 CP samples were analyzed by ESI-IM-HRMS. Among the obtained high-resolution MS signals (Figure 4), it was inferred that there might be particular PMFs in *Citrus* species at *m/z* 343.1168, *m/z* 373.1271, *m/z* 403.1381, *m/z* 433.1484 with a mass deviation within 5 ppm. Given the fact that mass spectrometry could only measure the mass-to-charge ratio, and it was limited to analyzing isobaric species, further observation on the 2D heat map of IMS found that there were two spots at *m/z* 373.1271, which meant that a pair of isomers were present. According to their accurate masses and CCS values, the isomers were identified as tangeretin and sinensetin. Similarly, the other two PMFs at *m/z* 403.1381 and *m/z* 433.1484 were identified as nobiletin and 3,5,6,7,8,2′,3′-heptamethoxyflavone, respectively.

In addition, we found that the mass spectra of GCP and CP were very similar, which did not assist in distinguishing between them, while it was tantamount to adding another dimension to the analysis when IM-MS was employed. For instance, there was a pair of isomers at *m/z* 373.1271, identified as tangerine and sinensetin, in samples based on the accurate mass and CCS values. Through further observation, we found that the relative proportion of these two isomers (tangerine and sinensetin) were different in GCP and CP. The ratio of tangerine and sinensetin in GCP was about 1:1.3, while it was about 1:2 in CP. What is more, the content of tangerine in GCP was about twice as much as in CP (Figure 4). It was confirmed that microorganisms could increase the contents of tangerine during the aging process of GCP [65]. Nevertheless, further systematic experiments are required to reveal the impact of various factors on the contents of tangerine and sinensetin, such as their genetic origin, growth environment, storage condition, and time of harvest. As mentioned above, it allowed the developed method as a reference to distinguish GCP from CP.

### 2.5. Distinction between GCP and CP Based on Multivariate Analysis

**Figure 5:**
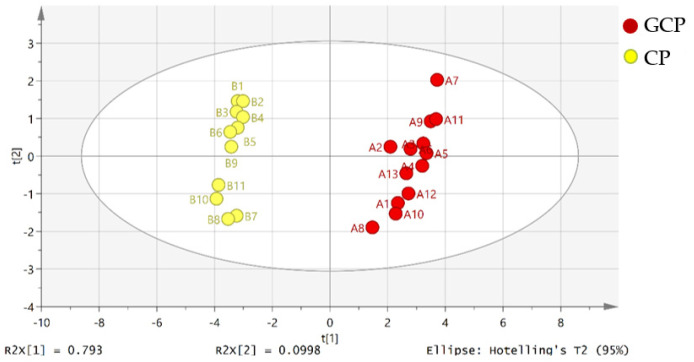
Score plot of the principal component analysis (PCA) of Guangchenpi (Group A) and Chenpi (Group B) samples. Red circles represent GCP samples, and yellow circles represent GCP samples.

The unsupervised principal component analysis (PCA) is a statistical method to mirror the trends of a dataset by a dimensional reduction in data [66]. Principal components (PCs) were searched from the input data matrix via maximal variance. We divided all of the samples into two groups and numbered them (group A were GCP samples and group B were CP samples). After the data were imported into SIMCA 14.1 (Umetrics, Malmö, Sweden), PCA was conducted. The score plot of PCA of Guangchenpi (Group A) and Chenpi (Group B) samples was shown in Figure 5. GCP samples in red were divided into a cluster and CP samples in yellow were grouped into another cluster. The variances were accounted for by the first principal component (PC1) and the second principal component (PC2), which were 79.3% and 9.9%, and the predictive ability of the model (Q^2^) was 78.7 %, indicating a successful model (Q^2^ ≥ 0.50).

**Figure 6:**
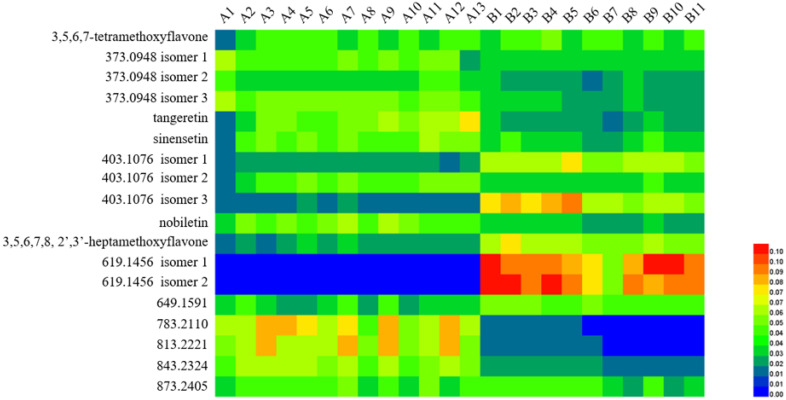
Heatmap of hierarchical cluster analysis (HCA) for analytes in Guangchenpi (Group A) and Chenpi (Group B) samples.

Hierarchical cluster analysis is a statistical analysis to find relatively homogeneous clusters of cases by measured characteristics. It was extensively adopted in species authentication and the quality control of traditional Chinese medicines [67]. To investigate the chemical variation based on MS data (Table S4 in Supplementary Materials), HCA was conducted by Heatmap Illustrator (HemI, v.1.0) [68]. All analyses were calibrated and normalized to generate a peak intensity matrix of the normalized percentage content for each chemical. The heat map was used to demonstrate the differences between the compounds GCP and CP (Figure 6). The samples were divided into two main clusters: samples of GCP and samples of CP. GCP samples were grouped, indicating that they had similar chemical profiles. The remaining samples were CP and were divided into another group, suggesting that CP had chemical profiles that were different from GCP.

## 3. Materials and Methods

### 3.1. Chemicals and Materials

HPLC grade methanol was purchased from Merck (Darmstadt, Germany). Deionized water was prepared by Milli-Q Advantage A 10 (Millipore Corp. Bedford, MA, USA). Tangerine and sinensetin (purity > 98%) were bought from Bide Pharmatech Ltd. (Shanghai, China). PTFE needle filters (diameter 13 mm, pore size 0.22 μm) were bought from ANPEL Laboratory Technologies Inc (Shanghai, China). In this study, a total of 24 batches of samples (13 batches of GCP and 11 batches of CP), were purchased from a Chinese manufacturer Shanghai Kangqiao Chinese Medicine Tablet Co., Ltd. (Shanghai, China).

GCP samples originated from Xinhui district (Jiangmen, China), and CP samples were derived from Bozhou (Bozhou, China). Information on samples is listed in Table S5 in the Supplementary Materials. The samples were preliminarily authenticated by Professor Tao Wu (Key Laboratory of Standardization of Chinese Medicines of Ministry of Education, Institute of Chinese Materia Medica, Shanghai University of Traditional Chinese Medicine, Shanghai, China) according to their morphological characteristics, and the voucher specimens were saved in Shanghai Institute of Organic Chemistry, Chinese Academy of Sciences, Shanghai.

### 3.2. Sample Preparation

Dried GCP and CP samples were grounded into powder by a grinding machine manufactured by Shanghai one bio Technology Co., LTD (Shanghai, China). Then, 0.2 g of individual sample powder was accurately weighed. Then, 5 mL of methanol was added to the sample and ultrasonic extraction was performed for 60 min at room temperature. After ultrasonic extraction, centrifugal separation was carried out for 5 min at 8000 rpm. GCP and CP samples were extracted by the process mentioned above. Then, each extract was filtered through 0.22 μm microporous membranes with a PTFE syringe. Finally, each filtrate was directly injected into the ESI-IM-HRMS system.

### 3.3. Electrospray Ionization–Ion Mobility–High-Resolution Mass Spectrometry Analysis (ESI-IM-HRMS)

**Figure 7:**
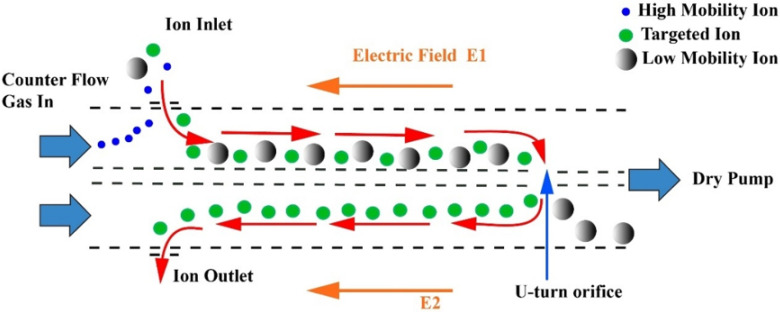
Schematic diagram of the U-shaped mobility analyzer. Red arrows indicate the ion path.

All experiments were carried out using an electrospray ionization quadrupole time-of-flight mass spectrometer LCMS 9030 (Shimadzu Corporation, Kyoto, Japan), equipped with the newly developed UMA device [57]. The UMA device adopted a technology named counter-flow that could greatly improve the resolution of IM. As shown in Figure 7, the UMA device mainly consisted of two parallel ion channels (CH1 and CH2) interlinked to form a mobility band-pass filter. The buffer gas was introduced into the two channels at the same speed in the opposite direction to the internal electric fields. When the ions emitted from the ion source entered the channels, they were influenced by converse forces, namely, the force of the counter-flow gas with a high speed and electric field force. In filter-scan mode, the high-mobility ions moved against the buffer gas flow in CH1 and the ions were eliminated upstream of CH1. The remaining ions continued to move along CH1 until turning 90° and entering the CH2 through the U-turn orifice. The low-mobility ions were removed downstream of CH2. Only targeted ions could pass through both CH1 and CH2 successfully. The name of this device is based on the trajectory of ions (U-shape).

The parameters of the UMA device were set as follows: mobility analysis mode was filter-scan mode; the electric field in CH1 was scanned from 1.0 V/mm to 4.0 V/mm, with a scan period of 1000 ms; the electric field difference between CH1 and CH2 was fixed as 0, and the countering gas flow rate was 1 L/min. Herein, high-purity nitrogen produced by the nitrogen generator was employed as the counter-flow gas, of which the temperature was maintained at 40 °C and the dew point was about −20~40 °C. Before CCS calculation, an external calibration was performed using an Agilent tune-mix ion solution (*m/z* 100 to 1200) adding 2 ng·mL^−1^ tetrahexylammonium (*m/z*: 354.4) and tetraoctylammonium (*m/z*: 466.5) in acetonitrile–water (*v/v* = 7:3, 0.1 % formic acid). The mass spectrometry settings were as follows: nebulizing gas flow, 3.0 L/min; drying gas flow, 10.0 L/min; heating gas flow, 10.0 L/min; interface voltage, 4.0 kV; interface current, 0.1mA; interface temperature, 100 °C; desolvation temperature, 160 °C; DL temperature, 250 °C; heat block temperature, 400 °C; flight tube temperature, 40 °C. In the positive mode, MS spectra were acquired from *m/z* 100 to 1000 in full scan mode. The collision energy was set at 30 eV with argon when MS/MS scans of five PMFs were performed. Before CCS measurements, the calibration of mass measurements was performed using sodium iodide. All IM-MS spectra were obtained with an auto-sampler by direct injection at a flow rate of 10 μL·min^−1^.

### 3.4. Data Analysis and Statistics

The data were acquired by UMA Data Processing (Shimadzu Corporation, Kyoto, Japan). Additionally, the CCS values were calculated according to an equation whose two main parameters were the central electric field and accurate mass. Ion mobility diagrams were drawn by origin 8 Pro (MicroCal Software, Northampton, MA). PCA was performed by SIMCA-P version 14.1 (Umetrics, Malmö, Sweden) and HCA was conducted by Heatmap Illustrator (HemI, v.1.0) to illustrate the distribution of compounds in GCP and CP and the results were presented as a dendrogram.

## 4. Conclusions

To summarize, a method for rapid discrimination between GCP and CP was established using ESI-IM-HRMS. It only took 1 min to perform a cycle of sample analysis and data acquisition, which saved time. With the combination of high-resolution ion mobility, the developed method showed excellent performance in separating isomers. In addition, the CCS values were measured as auxiliary parameters to characterize and identify the compounds in samples. Based on the above advantages, ESI-IM-HRMS was effectively applied to analyze GCP and CP samples. The results of multivariate analysis showed that GCP and CP were divided into two clusters. The method was validated as reliable and efficient for distinguishing between GCP and CP, providing a supplement for the identification of traditional Chinese medicines. Further work, which is establishing more convenient and applicable analytical methods for distinguishing between GCP and CP is underway.

## Figures and Tables

**Table 1:** Fragment ions of polymethoxylated flavones measured by ESI-IM-HRMS in the positive mode.

Compounds	[M + H]^+^ (Error, ppm)	Fragment Ions *m/z* (%) (Error, ppm)	Other Ions
3,5,6,7-tetramethoxyflavone	343.1168 (−2.3)	328.0931 (14) [M + H-CH_3_]^+^ (−3.0), 313.0699 (100) [M + H-C_2_H_6_]^+^ (−2.6)	299.0905 (5), 282.0877 (10)
tangeretin	373.1269 (−3.5)	358.1033(3) [M + H-CH_3_]^+^ (−3.9), 343.0802 (100) [M + H-C_2_H_6_]^+^ (−2.9), 328.0565(16) [M + H-C_3_H_9_]^+^ (−3.0), 315.085 (3) [M + H-C_2_H_6_-CO]^+^ (−2.9)	357.0958 (32), 325.0694 (7), 300.0616 (19), 297.0745 (10), 271.0588 (12), 183.0278 (8)
sinensetin	373.1271 (−2.9)	358.1036(5) [M + H-CH_3_]^+^ (−3.1), 343.0802 (100) [M + H-C_2_H_6_]^+^ (−2.9), 315.0852 (49) [M + H-C_2_H_6_-CO]^+^ (−3.5)	357.0958 (32), 339.0852 (22), 329.1009 (34), 327.0489 (14), 153.0173 (11), 151.0380 (13)
nobiletin	403.1381 (−1.5)	373.0913 (100) [M + H-C_2_H_6_]^+^ (−1.3), 358.0677 (10) [M + H-C_3_H_9_]^+^ (−1.7), 355.0805 (7) [M + H-C_2_H_6_-H_2_O]^+^ (−2.0), 345.0962 (6) [M + H-C_2_H_6_-CO]^+^ (−2.0), 330.0729 (10) [M + H-C_3_H_9_-CO]^+^(−1.5), 211.0230 (7) [^1,3^A^+^−C_2_H_6_] (−3.3), 163.0746 (1) [^1,3^B^+^] (−4.9)	327.0857 (16), 301.0699 (6), 189.0542 (4), 327.0857 (16), 301.0699 (6), 189.0542 (4)
3,5,6,7,8,2′,3′-heptamethoxyflavone	433.1484 (−2.1)	403.1019 (100) [M + H-C_2_H_6_]^+^ (−1.2), 388.0783(8) [M + H-C_3_H_9_]^+^ (−1.5), 360.0833 (5) [M + H-C_3_H_9_-CO]^+^ (−1.9), 345.0599 (5) [M + H-C_4_H_12_-CO]^+^ (−1.7), 211.0229(2) [^1,3^A^+^-C_2_H_6_] (−3.3)	387.0704 (4), 385.0911 (8), 373.0549 (6), 360.0833 (5)

**Table 2:** The collision cross-section (CCS) values were measured in samples.

No.	Measured *m/z*	Ion	CCS Values of Samples (Å^2^)
1	343.1168	[M + H]^+^	171.2 ± 1.7
2	373.0948	[M + H]^+^	183.0 ± 0.4
3	373.0948	[M + H]^+^	188.1 ± 0.4
4	373.0948	[M + H]^+^	193.8 ± 0.5
5	373.1271	[M + H]^+^	184.3 ± 0.5
6	373.1271	[M + H]^+^	187.7 ± 0.3
7	403.1076	[M + H]^+^	189.8 ± 0.1
8	403.1076	[M + H]^+^	193.0 ± 0.4
9	403.1076	[M + H]^+^	201.0 ± 0.4
10	403.1381	[M + H]^+^	193.0 ± 0.4
11	433.1484	[M + H]^+^	200.5 ± 0.4
12	619.1456	[M + H]^+^	225.6 ± 0.1
13	619.1456	[M + H]^+^	232.1 ± 0.3
14	649.1591	[M + H]^+^	230.1 ± 0.1
15	783.2110	[M + H]^+^	259.3 ± 0.8
16	813.2221	[M + H]^+^	263.4 ± 0.1
17	843.2324	[M + H]^+^	268.8 ± 0.3
18	873.2405	[M + H]^+^	274.6 ± 0.1

**Table 3:** The collision cross section (CCS) values of samples of [TAN + H]^+^ and [SIN + H]^+^ in quintuples, found in the literature.

Compounds	Ion	CCS Values of Samples by UMA-MS (Å^2^)	CCS Values of Standards by DTIMS (Å^2^)
Tangeretin	[TAN + H]^+^	184.3 ± 0.5	183.9 ± 1.1
Sinensetin	[SIN + H]^+^	187.7 ± 0.3	187.8 ± 1.0

## Data Availability

The data presented in this study are available on request from the corresponding author.

## Supplementary Materials

The following are available online, Figure S1: Optimization of sample extraction: (a) extraction solvent, (b) volume of methanol, (c) ultrasonic extraction time, Figure S2: Effect of different electric field ranges on separation of tangeretin and sinensetin:(a) E:2.1–2.6 V/mm; (b) E:2–3 V/mm; (c) E:1–4 V/mm; (d) E:1–6 V/mm, Figure S3: Effect of the rate of counter-flow gas (1.0 L/min) on separation of tangeretin and sinensetin, Figure S4: MS spectra and UMA spectra of PMFs in CP and GCP (a) tangeretin and sinensetin, (b) nobiletin, (c) 3,5,6,7,8,2′,3′-heptamethoxyflavone, Figure S5: MS spectra (top) and drift tube spectra (bottom) of tangerine and sinensetin by drift tube ion mobility: (a) tangerine, (b) sinensetin, (c) mixed tangerine and sinensetin, Table S1: The repeatability of ESI-IM-HRMS system (*n* = 6), Table S2: The reproducibility of ESI-IM-HRMS system(*n* = 9), Table S3: The collision cross section (CCS) values of samples in quintuples, Table S4: Mass spectra signals and intensity of each sample, Table S5: The information of GCP and CP samples.

## Sample Availability

Samples of tangerine and sinensetin are available from the authors.

## Abbreviations

IMS	ion mobility spectrometry
UMA	U-shaped mobility analyzer
HRMS	high-resolution mass spectrometry
DTIMS	drift tube ion mobility spectrometry
TW-IMS	traveling wave ion mobility spectrometry
TIMS	trapped ion mobility spectrometry
cIMS	cyclic ion mobility spectrometry
CCS	ollision cross-section
Q-TOF MS	quadrupole time of flight mass spectrometry
PMFs	polymethoxylated flavones
PCA	principal component analysis
HCA	hierarchical cluster analysis
CRP	*Citri Reticulatae* Pericarpium
CP	Chenpi
GCP	Guangchenpi *C. reticulata* ‘Chachi.
