# Supplementary material for: Rapid Discrimination of Citrus reticulata ‘Chachi’ by Electrospray Ionization–Ion Mobility–High-Resolution Mass Spectrometry
Source: Molecules. 2021 Nov 20;26(22):7015. doi: 10.3390/molecules26227015 (PMC8622672; doi:10.3390/molecules26227015)
Supplement: Supplementary file 1 [file molecules-26-07015-s001.zip › molecules-1443156-supplementary.pdf]

Supplementary material

1 Optimization of sample extraction..2

2 Effect of different electric fields on separation of tangeretin and sinensetin 4

3 Effect of gas flow on separation of tangeretin and sinensetin 5

4 MS spectra and UMA spectra of polymethoxylated flavones in CP and GCP...........6

5 Tangerine and sinensetin were analyzed by drift tube ion mobility spectrometry. 6

6 The possible fragmentation pattern of nobiletin. 8

7 MS^2 spectra of five polymethoxylated flavones. 9

8 The repeatability and reproducibility of ESI-IM-Q-TOF MS system. 13

9 The collision cross section (CCS) value measurements 14

10 The data of signals and ion mobility intensity in each sample 15

11 Information of GCP and CP samples...18

1 Optimization of sample extraction

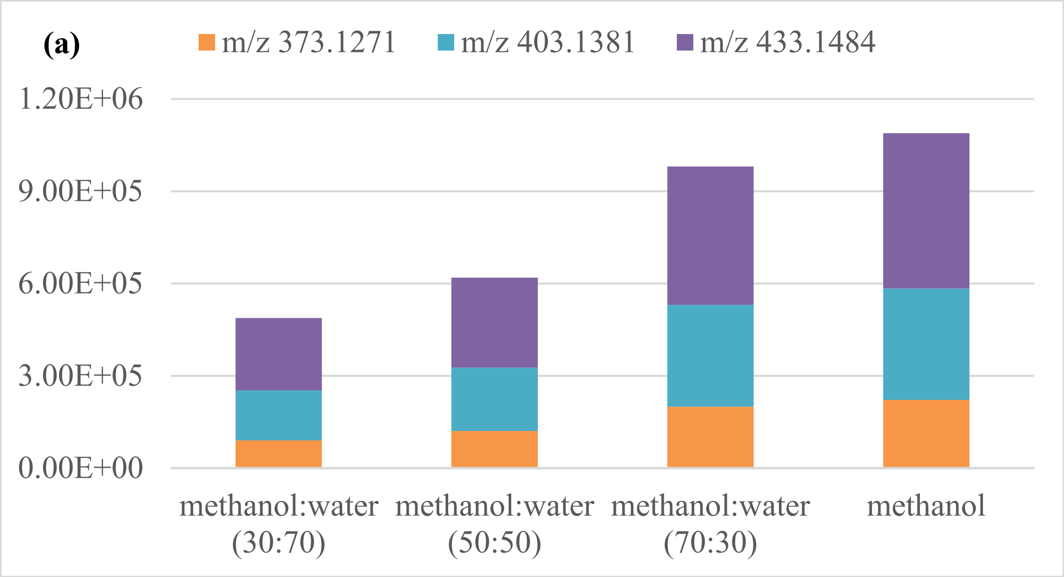

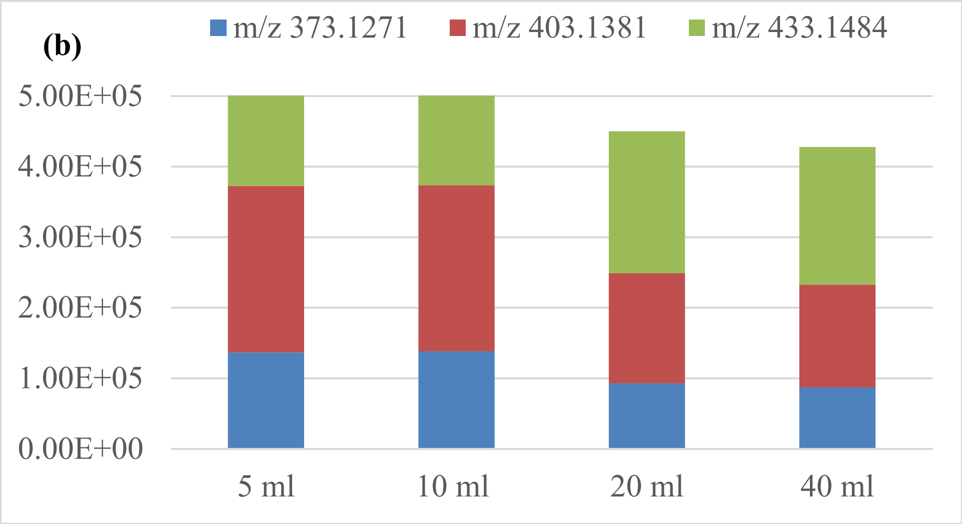

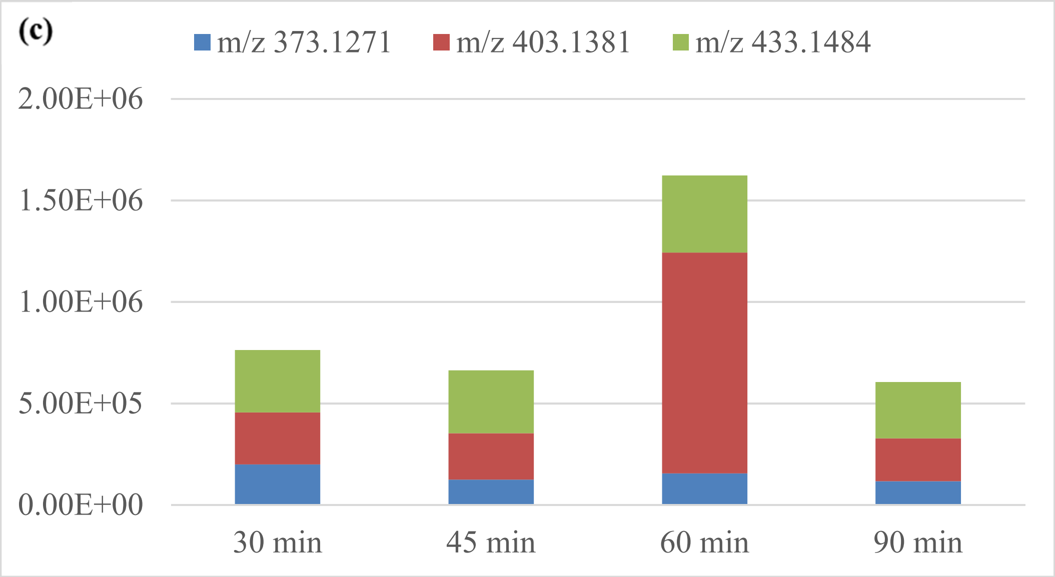

Figure S1. Optimization of sample extraction:(a) extraction solvent, (b) volume of methanol, (c) ultrasonic extraction time.

2 Effect of different electric fields on separation of tangeretin and sinensetin

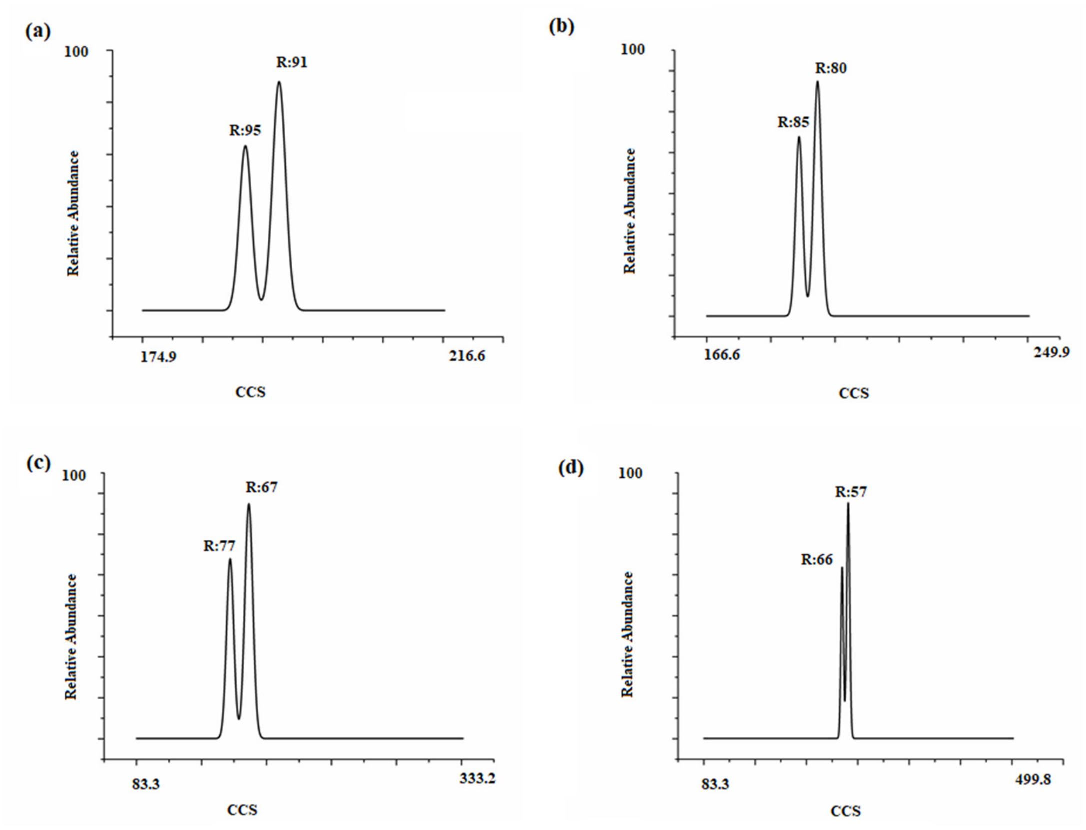

Figure S2. Effect of different electric field ranges on separation of tangeretin and sinensetin:(a) E:2.1-2.6 V/mm; (b) E:2-3 V/mm; (c) E:1-4 V/mm; (d) E:1-6 V/mm.

3 Effect of gas flow on separation of tangeretin and sinensetin

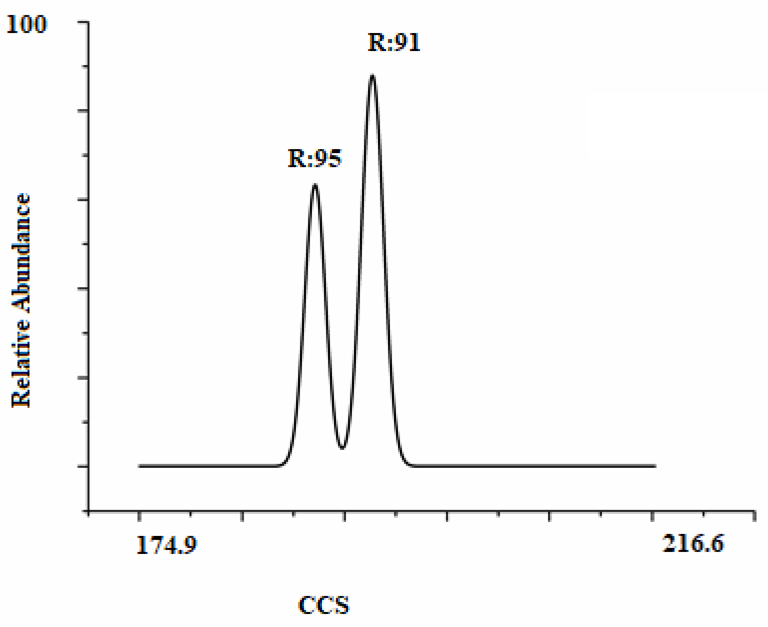

Figure S3. Effect of the rate of counter-flow gas (1.0 L/min) on separation of tangeretin and sinensetin

4 MS spectra and UMA spectra of polymethoxylated flavones in CP and GCP

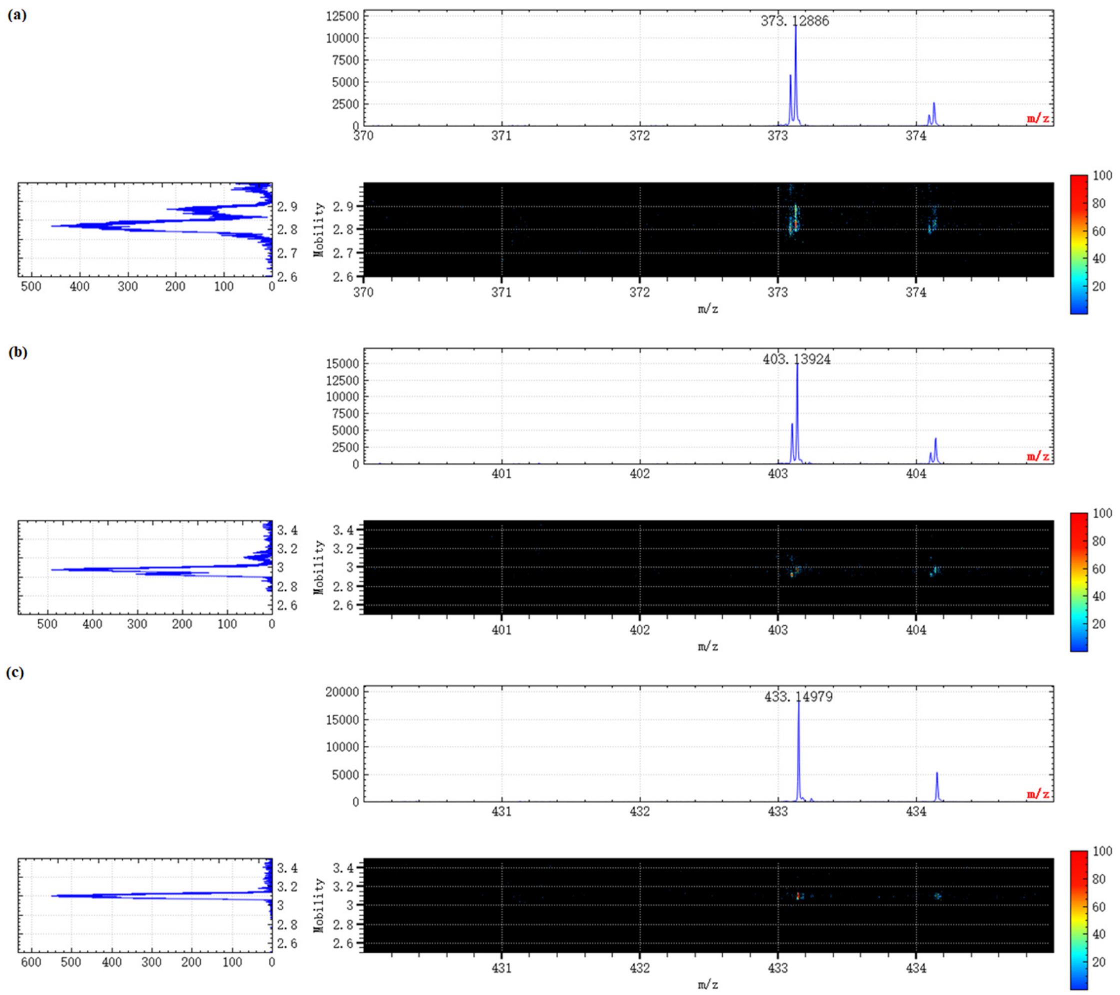

Figure S4. MS spectrum and UMA spectra of polymethoxylated flavones in CP and GCP (a) tangeretin and sinensetin, (b) nobiletin, (c) 3,5,6,7,8, 2’,3’-heptamethoxyflavone.

5 Tangerine and sinensetin were analyzed by drift tube ion mobility spectrometry.

An ion-mobility quadrupole time-of-flight mass spectrometer (IM-Q-TOF MS, Agilent Technologies, Santa Clara, CA) was employed. The instrument set is as follows: the gas temperature was 300 °C, drying gas was 5 L/min, sheath gas temperature was 350 °C, sheath gas flow was 11 L/min, nozzle voltage was 2000V, nitrogen as the IM drift gas was maintained at around 4 Torr and 25 °C, the drift voltage was set as 1200

V. The resolution of IM was about 60 td/Δt (drift time, full width at half-maximum).

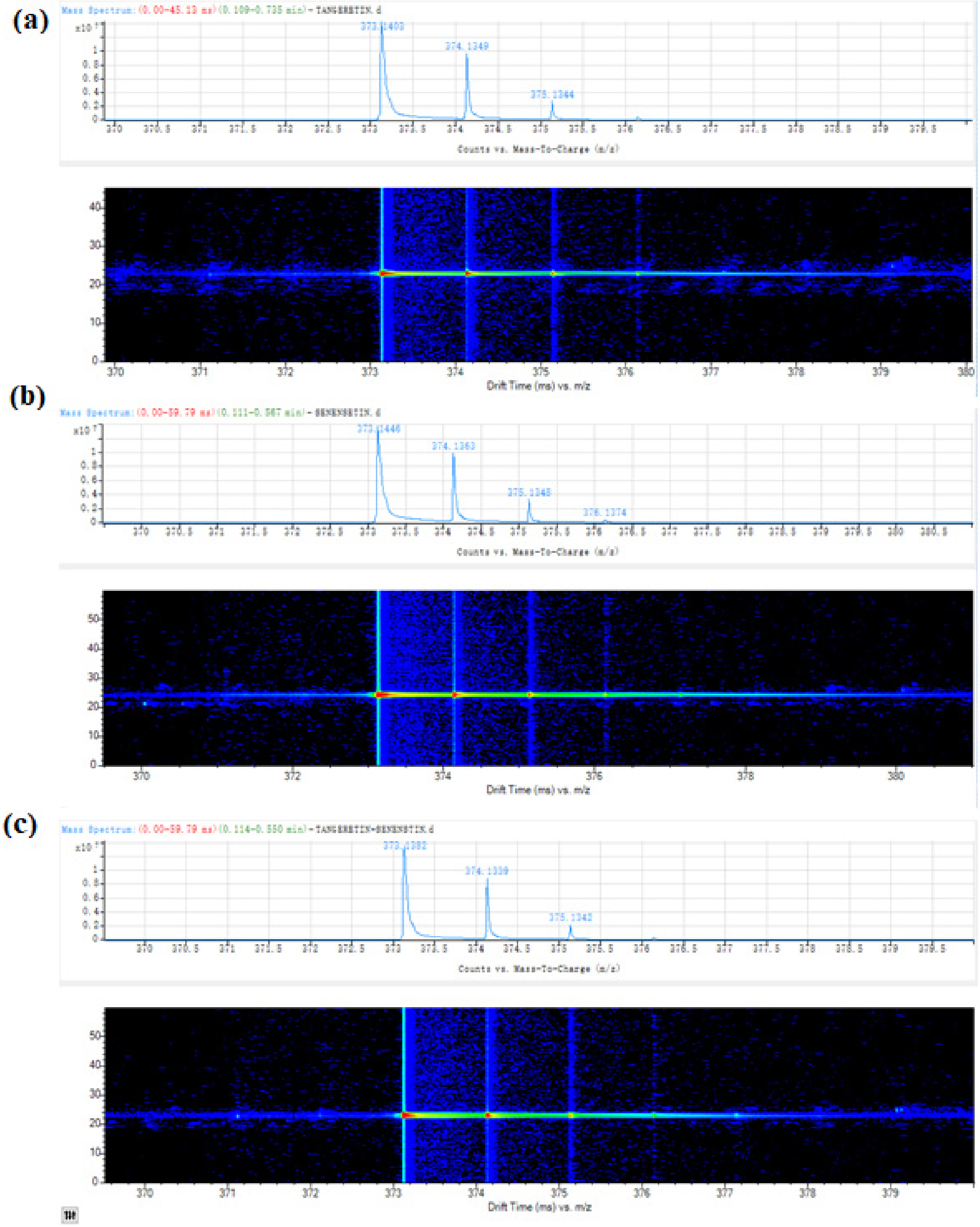

Figure S5. MS spectra (top) and ion mobility spectra (bottom) of tangerine and sinensetin by drift tube ion mobility spectrometry: (a) tangerine, (b) sinensetin, (c) mixed tangerine and sinensetin.

6 The possible fragmentation pattern of nobiletin.

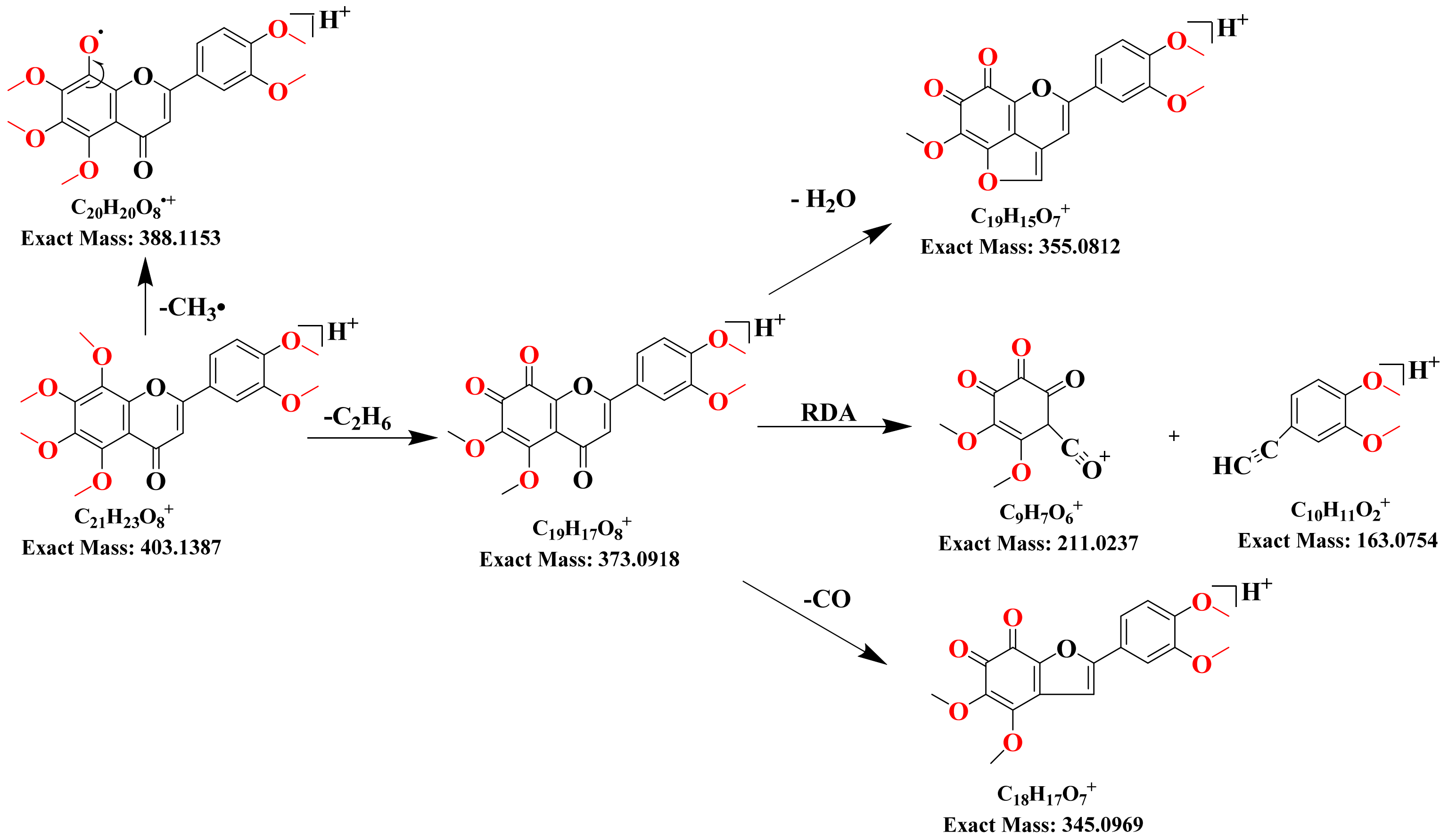

Figure S6. The possible fragmentation pattern of nobiletin.

7 MS^2 spectra of five polymethoxylated flavones.

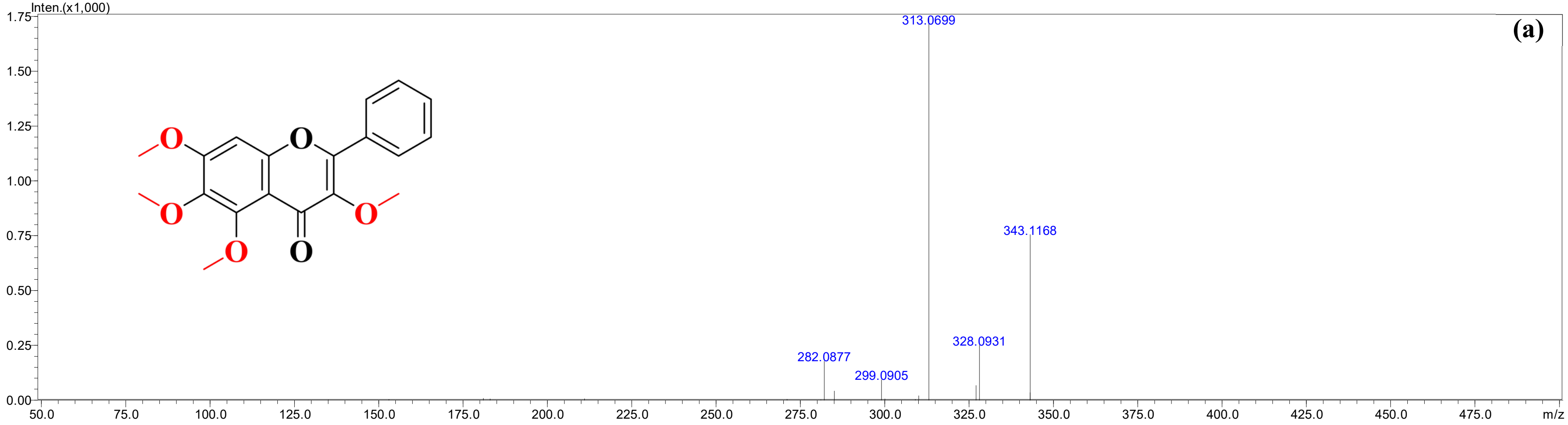

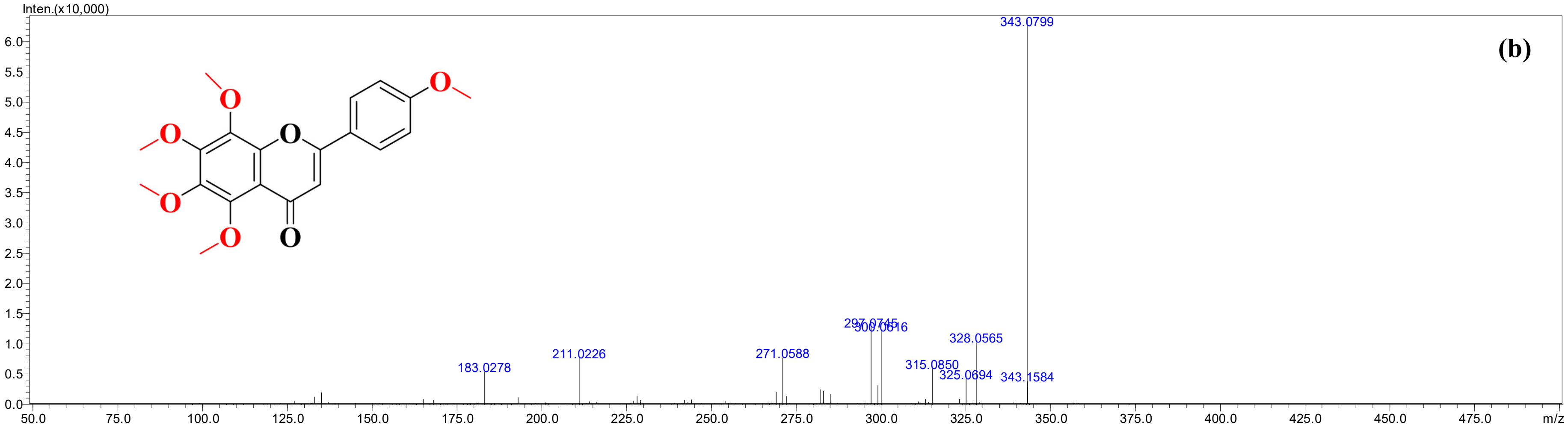

Inten.(x10,000)
(b)
343.0799
297.0745
300.0616
328.0565
315.0850
325.0694
343.1584
271.0588
211.0226
183.0278
m/z

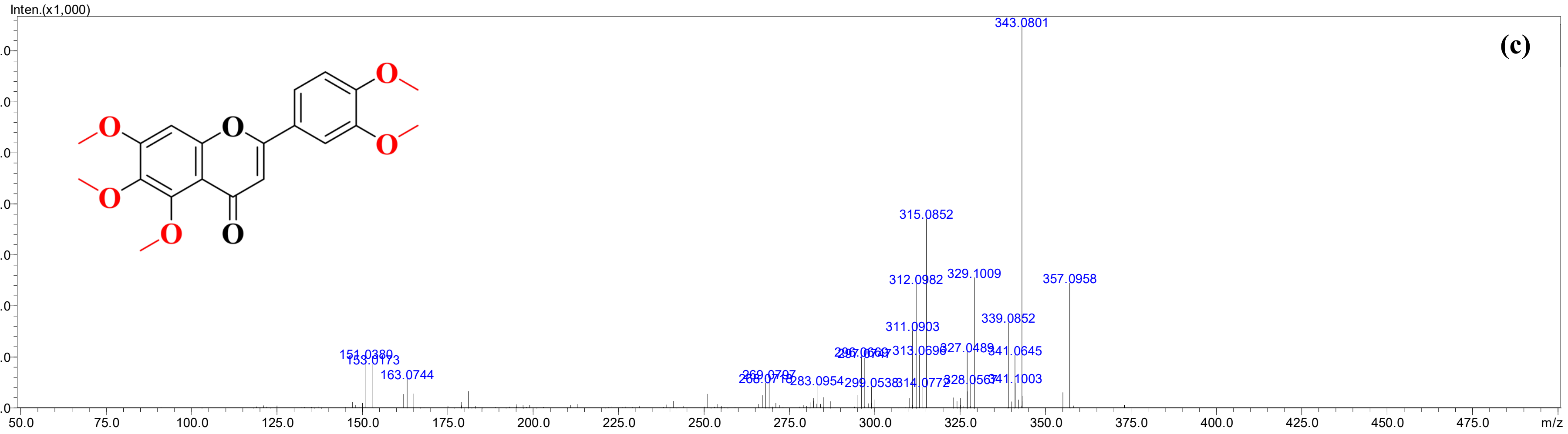

Inten.(x1,000)
(c)
343.0801
315.0852
312.0982
329.1009
357.0958
311.0903
339.0852
151.0380
153.0173
163.0744
313.0696
327.0489
341.0645
283.0954
299.0538
314.0772
328.0567
341.1003
m/z

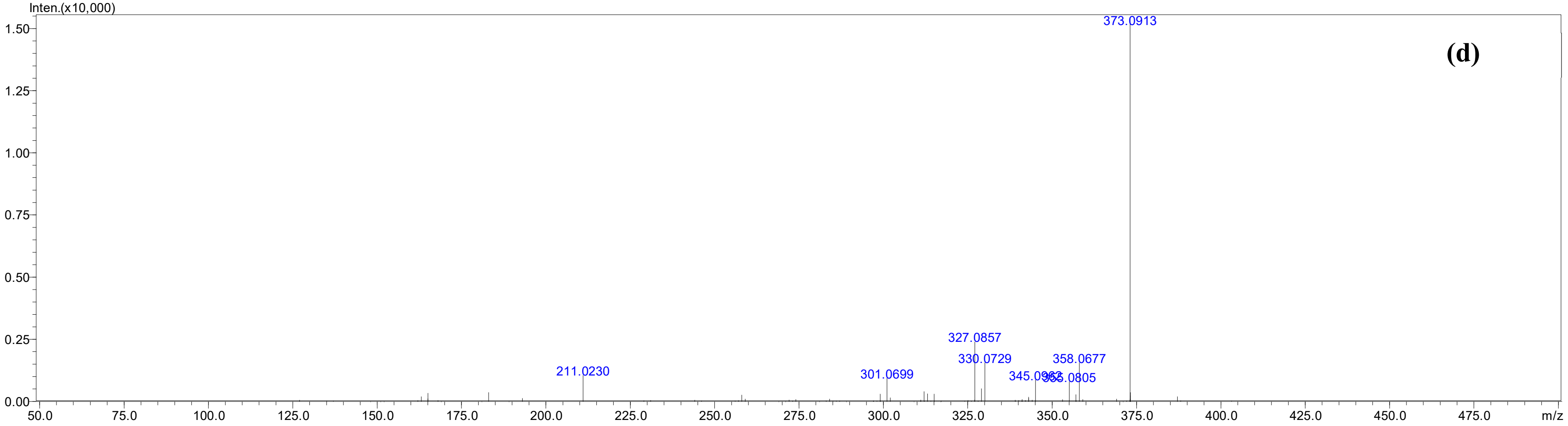
Inten.(x10,000)
1.50
1.25
1.00
0.75
0.50
0.25
0.00
373.0913
(d)
211.0230
301.0699
327.0857
330.0729
345.0962
355.0805
358.0677
50.0
75.0
100.0
125.0
150.0
175.0
200.0
225.0
250.0
275.0
300.0
325.0
350.0
375.0
400.0
425.0
450.0
475.0
m/z

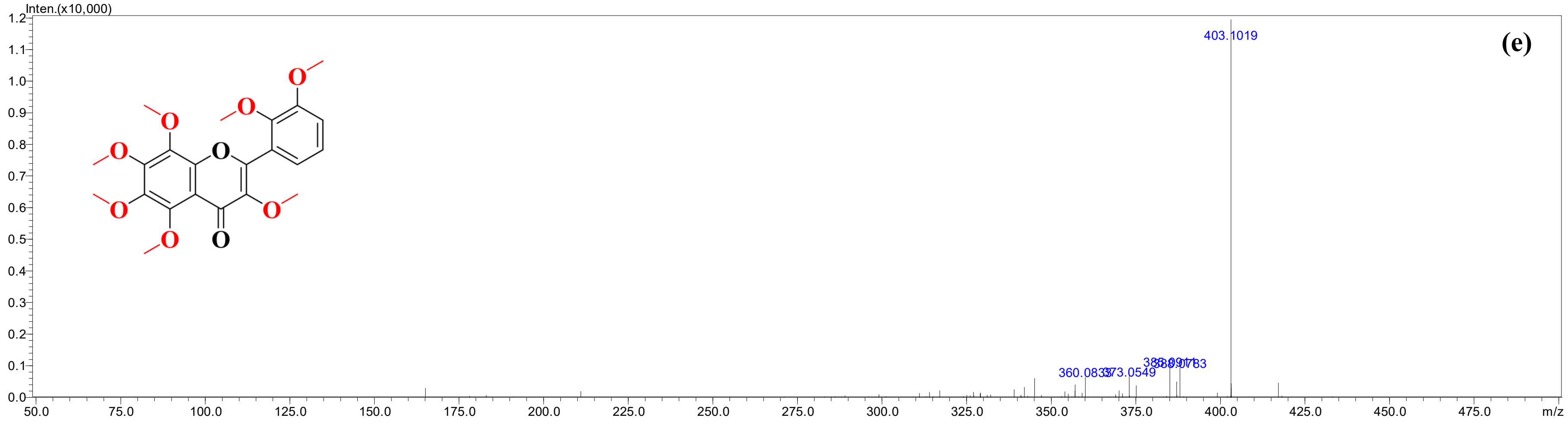

Figure S7. MS^2 spectra of five polymethoxylated flavones. (a) 3,5,6,7-tetramethoxyflavone; (b) tangeretin; (c) sinensetin; (d) nobiletin,; (e) 3,5,6,7,8, 2',3'-heptamethoxyflavone.

8 The repeatability and reproducibility of ESI-IM-Q-TOF MS system.

Table S1. The repeatability of ESI-IM-Q-TOF MS system (n=6)

	1	2	3	4	5	6	RSD (%)
Intensity	652	669	671	650	637	642	1.9

Table S2. The reproducibility of ESI-IM-Q-TOF MS system (n=9)

	1-1	1-2	1-3	2-1	2-2	2-3	3-1	3-2	3-3	RSD (%)
Intensity	853	870	840	821	814	815	825	804	853	4.9

9 The collision cross section (CCS) value measurements

Table S3. The collision cross section (CCS) values of samples in quintuples

No.	m/z	CCS values of sample1	CCS values of sample2	CCS values of sample3	CCS values of sample4	CCS values of sample5	Mean±RSD（%）
1	343.1168	172.7	173.1	168.3	171.0	171.1	172.2 ±1.7
2	373.0948	182.7	183.2	183.4	183.3	182.3	183.0 ±0.4
3	373.0948	187.4	188.5	188.4	188.3	187.8	188.1 ±0.4
4	373.0948	192.9	194.6	193.9	193.8	193.8	193.8 ±0.5
5	373.1271	183.3	184.7	184.6	184.5	184.4	184.3 ±0.5
6	373.1271	187.2	188.0	188.0	187.7	187.6	187.7 ±0.3
7	403.1076	189.8	189.9	189.7	189.8	189.5	189.8 ±0.1
8	403.1076	192.2	193.3	193.2	193.2	193.0	193.0 ±0.4
9	403.1076	200.3	201.2	201.3	201.2	201.1	201.0 ±0.4
10	403.1381	192.3	193.4	193.3	193.3	193.0	193.0 ±0.4
11	433.1484	199.6	200.7	200.8	200.6	200.6	200.5 ±0.4
12	619.1456	225.7	225.7	225.5	225.7	225.5	225.6 ±0.1
13	619.1456	232.4	232.2	232.4	232.0	231.6	232.1 ±0.3
14	649.1591	230.0	230.1	230.2	230.2	230.0	230.1 ±0.1
15	783.2110	260.2	258.7	260.3	258.6	258.6	259.3 ±0.8
16	813.2221	263.4	263.5	263.4	263.4	263.3	263.4 ±0.1
17	843.2324	268.9	268.3	269.0	269.0	268.9	268.8 ±0.3
18	873.2405	274.4	274.6	274.7	274.7	274.7	274.6 ±0.1

10 The data of signals and ion mobility intensity in each sample

Table S4. Mass signals and ion mobility intensity of each sample

No. \ m/z	343.1168	373.0948			373.1275		403.1076			403.1383	433.1184	619.1456		649.1591	783.2110	813.2221	843.2324	873.2405
**A1**	31	812	437	407	49	59	109	123	25	771	321	0	0	514	608	603	419	200
**A2**	83	537	315	283	113	184	240	244	33	997	394	0	0	690	555	670	549	215
**A3**	54	593	330	341	148	213	244	279	30	841	293	0	0	516	752	921	595	243
**A4**	92	608	302	336	150	177	272	301	38	1129	446	0	0	360	760	734	569	223
**A5**	87	632	373	371	141	232	300	328	54	941	549	0	0	369	647	717	554	242
**A6**	97	560	332	322	141	205	249	285	45	1128	471	0	0	581	612	751	526	217
**A7**	78	667	364	348	147	219	304	351	53	1267	524	0	0	620	653	914	594	290
**A8**	103	642	339	343	154	201	250	311	36	905	433	0	0	449	444	578	416	167
**A9**	69	660	356	338	184	210	231	301	32	1225	437	0	0	726	764	897	614	223
**A10**	86	614	318	282	168	192	222	301	35	1140	444	0	0	420	510	595	415	158
**A12**	84	747	435	361	191	267	293	350	38	983	503	0	0	559	628	703	518	257
**A13**	96	756	442	367	197	219	194	319	27	904	380	0	0	583	801	894	531	192
**A14**	104	310	340	310	205	216	231	342	34	884	348	0	0	456	567	635	468	215
**B1**	83	515	245	229	93	160	686	194	183	680	1177	473	387	860	109	179	229	240
**B2**	95	479	269	205	82	170	719	245	202	691	1232	430	361	805	124	161	248	248
**B3**	105	478	238	208	80	160	663	230	176	661	1137	436	350	890	102	217	234	226
**B4**	113	461	234	203	84	161	693	233	212	700	1150	438	358	707	99	164	252	242
**B5**	82	435	205	166	68	135	746	218	217	636	1111	366	324	718	125	163	241	233
**B6**	95	403	176	159	70	118	561	194	162	575	1010	322	248	790	79	124	209	232
**B7**	94	403	206	151	54	106	618	199	154	587	980	252	189	611	50	81	121	155

**B8**	7	519	303	229	66	140	628	208	130	502	1010	400	325	726	35	72	103	133
**B9**	10	437	222	163	112	208	672	302	159	655	1091	451	306	731	45	101	168	205
**B10**	7	427	222	155	75	146	654	215	157	559	1021	462	329	740	41	70	118	143
**B11**	13	417	191	151	72	150	551	195	131	566	963	423	329	725	42	90	150	166

11 Information of GCP and CP samples

Table S5 Information of GCP and CP sample.

Sample No.	Origins	Species
GCP A1	Xinhui, Jiangmen, China	*C. reticulata* ‘Chachi’
GCP A2	Xinhui, Jiangmen, China	*C. reticulata* ‘Chachi’
GCP A3	Xinhui, Jiangmen, China	*C. reticulata* ‘Chachi’
GCP A4	Xinhui, Jiangmen, China	*C. reticulata* ‘Chachi’
GCP A5	Xinhui, Jiangmen, China	*C. reticulata* ‘Chachi’
GCP A6	Xinhui, Jiangmen, China	*C. reticulata* ‘Chachi’
GCP A7	Xinhui, Jiangmen, China	*C. reticulata* ‘Chachi’
GCP A8	Xinhui, Jiangmen, China	*C. reticulata* ‘Chachi’
GCP A9	Xinhui, Jiangmen, China	*C. reticulata* ‘Chachi’
GCP A10	Xinhui, Jiangmen, China	*C. reticulata* ‘Chachi’
GCP A11	Xinhui, Jiangmen, China	*C. reticulata* ‘Chachi’
GCP A12	Xinhui, Jiangmen, China	*C. reticulata* ‘Chachi’
GCP A13	Xinhui, Jiangmen, China	*C. reticulata* ‘Chachi’
CP B1	Bozhou, China	*C. reticulatae* pericarpium
CP B2	Bozhou, China	*C. reticulatae* pericarpium
CP B3	Bozhou, China	*C. reticulatae* pericarpium
CP B4	Bozhou, China	*C. reticulatae* pericarpium
CP B5	Bozhou, China	*C. reticulatae* pericarpium
CP B6	Bozhou, China	*C. reticulatae* pericarpium
CP B7	Bozhou, China	*C. reticulatae* pericarpium
CP B8	Bozhou, China	*C. reticulatae* pericarpium
CP B9	Bozhou, China	*C. reticulatae* pericarpium
CP B10	Bozhou, China	*C. reticulatae* pericarpium
CP B11	Bozhou, China	*C. reticulatae* pericarpium
